# The Negative Role of Proton Insertion on the Lifetime of Vanadium‐Based Aqueous Zinc Batteries

**DOI:** 10.1002/advs.202414762

**Published:** 2025-01-31

**Authors:** Chaoqiong Zhu, Limin Zheng, Hao Ruan, Meng Xiao, Meng Ye, Ting Chen, Fang Wan, Xiaodong Guo

**Affiliations:** ^1^ School of Chemical Engineering Sichuan University Chengdu 610065 P. R. China; ^2^ Institute for Advanced Study Chengdu University Chengdu 610106 P. R. China

**Keywords:** aqueous zinc batteries, coordination environment of water, proton, vanadium dissolution

## Abstract

Vanadium oxides are attracted cathodes for aqueous zinc batteries owing to their high capacity. However, the limited cyclability of vanadium‐based oxide cathodes, especially at low current densities, impedes their practical application. Here, it is revealed that proton insertion is responsible for the limited lifetime of vanadium oxides. Proton insertion promotes the dissolution of vanadium oxides, deteriorating electrochemical performance. Propylene carbonate (PC) is introduced into Zn(CF_3_SO_3_)_2_ electrolyte to regulate the coordination environment of water, forming PC‐coordinated Zn^2+^ solvation structure and [H_2_O‐CF_3_SO_3_^−^‐PC] complex. The optimized coordination environment of water weakens the adsorption energy between water molecules and vanadium oxides, inhibiting proton insertion. As a result, vanadium‐based oxides cathode without proton insertion can maintain the stability of crystal structure and avoid the dissolution of V. Taking CaV_8_O_20_·nH_2_O as cathode, Zn||CaV_8_O_20_·nH_2_O battery without proton insertion performs enhanced cycling performance. This work not only reveals the negative effect of proton insertion on the lifetime of vanadium‐based oxides cathode but also provides an effective strategy to modulate proton insertion.

## Graphical abstract

It is demonstrated that proton insertion accelerates the dissolution of vanadium oxides to form electrochemically inactive Zn_3_V_2_O_7_(OH)_2_·nH_2_O, deteriorating the electrochemical performance. The proton insertion is inhibited by regulating coordination environment of water in electrolyte to weaken the adsorption energy between water molecules and vanadium oxides. As a result, the battery without proton insertion displays enhanced cycling performance.

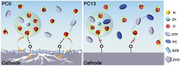

## 1 Introduction

Future energy mixing with a high share of intermittent renewable requires large‐scale energy storage devices to balance demand and supply.^[^ ^1^ ^]^ Aqueous rechargeable batteries are promising alternatives for large‐scale energy storage due to the utilization of low‐cost and safe aqueous electrolytes.^[^ ^2^ ^]^ Moreover, aqueous rechargeable batteries involve simple assembly conditions without rigorous management of moisture and oxygen content in ambiance.^[^ ^3^ ^]^ Among various aqueous batteries, Zn‐based systems are particularly attractive, owing to the advantages of Zn anode involving low redox potential (−0.76 V vs standard hydrogen electrode), high theoretical capacity (820 mAh g^−1^ and 5851 mAh cm^−3^), earth abundance and cost effectiveness.^[^ ^4^ ^]^

Since the Zn||Zn_0.25_V_2_O_5_·nH_2_O system was reported by Nazar's group, a series of vanadium‐based oxide cathodes have been reported for aqueous Zn batteries.^[^ ^5^ ^]^ However, vanadium oxides suffer from severe capacity degradation over cycling, especially cycling at low current density (i.e., <1 A g^−1^).^[^ ^6^ ^]^ The degradation mechanism being relating to the dissolution of vanadium has been demonstrated, while the negative role of proton insertion on it is ignored easily. Recent studies have found that nearly half capacity of vanadium oxides relies on proton (de)intercalation in neutral electrolytes.^[^ ^7^ ^]^ During discharging, proton intercalation consumes electrolytes to form the basic zinc salt (BZS, such as Zn_x_(CF_3_SO_3_)_y_(OH)_zx‐y_·nH_2_O) depositing on the electrode surface.^[^ ^8^ ^]^ During charging, the deintercalation of the proton decreasing the local pH at the electrode interface causes the dissolution of BZS.^[^ ^9^ ^]^ Noteworthy, non 100% reversible deintercalation of protons would lead to the accumulation of insulating BZS on the electrode surface, which could increase the diffusion energy barrier of Zn^2+^/H^+^.^[^ ^10^ ^]^ Moreover, the deposition and dissolution of BZS leads to large volume changes of cathode during charge/discharge process, exacerbating the peeling of active material. The above review clearly indicates that the electrochemical behavior of proton insertion for vanadium‐based oxides cathode is not friendly to maintaining integrated crystal structure and stable electrochemical performance. Especially at low current density, the volume change and accumulation of by‐products would more severely under deep charging and discharging. As a result, suppressing proton insertion would be an effective strategy to improve the cycling performance of vanadium oxides.

**Figure 1:**
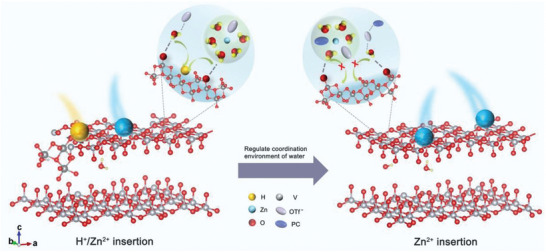
Schematic illustration of PC regulating coordination environment of water in electrolyte to suppress proton insertion.

Proton insertion undergoes the adsorption of water molecules on the surface of vanadium oxides and then the ionization of adsorbed water molecules to produce protons.^[^ ^9^, ^11^ ^]^ It can be seen that the key point to suppress proton insertion is to adjust the adsorption energy of water molecules on the surface of vanadium oxides. Herein, we introduce propylene carbonate (PC) into Zn(CF_3_SO_3_)_2_ (Zn(OTf)_2_) electrolyte to regulate water coordination environment (**Figure** **1**). PC molecules participating in Zn^2+^ solvation structure and forming [H_2_O‐OTf^−^‐PC] complex increase the bonding energy barrier between water molecules and terminal oxygen of vanadium oxides. Taking CaV_8_O_20_·nH_2_O (CVO) as the cathode, Zn||CVO battery with PC‐optimized electrolyte displays only Zn^2+^ insertion. Owing to the suppression of proton insertion, the by‐product (Zn_3_V_2_O_7_(OH)_2_·nH_2_O) from vanadium dissolution is not detected on electrode surface even after 100 cycles. As a result, Zn||CVO battery with only Zn^2+^ insertion performs better cycling performance than that with Zn^2+^/H^+^ co‐insertion.

## 2 Results and Discussion

### 2.1 The Electrochemical Behavior of Proton Insertion

**Figure 2:**
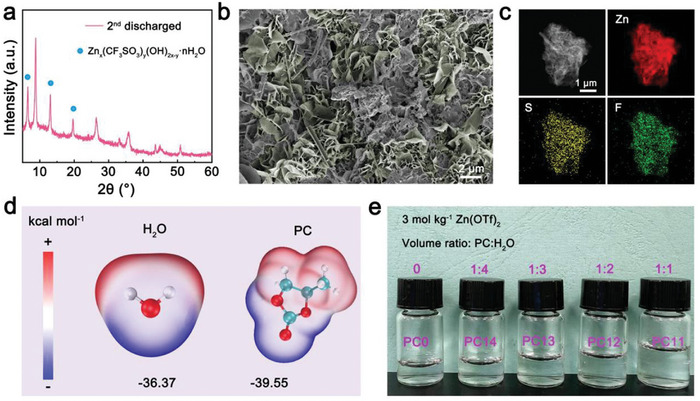
The structural and morphological analyses of CVO after 2 cycles. The XRD patters a) and SEM image b) of discharged CVO electrode after 2 cycles at 0.2 A g^−1^. c) The corresponding TEM element mapping images of discharged CVO electrode after 2 cycles at 0.2 A g^−1^. d) The surface electrostatic potential (ESP) of PC molecules and H_2_O molecules. e) The optical photographs of electrolytes with different volume ratios between PC and H_2_O.

CVO was prepared by a one‐step hydrothermal route. As shown in Figure S1 (Supporting Information), the CVO powder exhibits nanorod morphology and the XRD pattern of CVO matches well with CaV_8_O_20_·nH_2_O (JCPDS#45‐1362). The XRD patterns of the discharged CVO electrode were detected after 2 cycles at 0.2 A g^−1^. Compared to the XRD of pristine CVO electrode, additional three well‐resolved peaks at 6.54°, 13.04°, 19.69° are observed and these peaks correspond with the BZS (Zn_x_(CF_3_SO_3_)_y_(OH)_2x‐y_·nH_2_O) phase (**Figure** **2**a; Figure S2a, Supporting Information).^[^ ^12^ ^]^ These diffraction peaks of BZS disappear at the charged state of CVO electrode (Figure S3, Supporting Information). The identity of BZS was also confirmed by scanning electronic microscopy (SEM) images and transmission electron microscopy (TEM) element mapping images. Except for the nanorod morphology for pristine CVO powder, numerous sheet‐like by‐products adhere to the surface of the discharged CVO electrode (Figure 2b; and Figure S2b, Supporting Information). The element mapping of these by‐products shows obvious S, F, and Zn signals accorded with the composition of BZS (Figure 2c; Figure S4, Supporting Information). Above all, the synthesized CVO exists the electrochemical behavior of proton insertion.

The previous reports speculated that there are two possible sources of proton: the dissolution of vanadium oxides and the ionization of water molecules in electrolyte.^[^ ^13^ ^]^ Vanadium oxides dissolving in water would form yellow VO_2_(OH)_2_^−^ solution. Therefore, we immerse CVO electrode into pure water and Zn(OTf)_2_ electrolyte (3 mol kg^−1^) to observe the dissolution of CVO. The pure water and Zn(OTf)_2_ electrolyte still remain transparent and the contents of vanadium in pure water and Zn(OTf)_2_ electrolytes are 7.93 and 1.67 ppm after 5 days (Figure S5a and Table S1, Supporting Information). Moreover, there is no impurity phase from the XRD patterns of the soaked electrodes (Figure S5b, Supporting Information). These results illustrate that CVO electrode has excellent structure stability to avoid V dissolution in Zn(OTf)_2_ electrolyte. These results also could verify that proton insertion of CVO may be directly from the ionization of water molecules in electrolyte instead of generating by the dissolution of CVO. Proton insertion undergoes the adsorption of water molecules on the surface of electrode which is closely to coordination environment of water molecules in electrolyte.^[^ ^9^, ^13^ ^]^ Hence, it would be an effective strategy to inhibit proton insertion via regulating the coordination environment of water in electrolyte. PC, as a common electrolyte additive, displays a similar surface electrostatic potential compared with water molecules, which indicates that PC molecules could replace water molecules into Zn^2+^ solvation structure after introducing PC into pure Zn(OTf)_2_ electrolyte (Figure 2d). As shown in Figure S6 (Supporting Information), PC with hydrophobic groups of ‐CH_3_ is immiscible with water. However, OTf^−^ anions have hydrophobic −CF_3_ group and hydrophilic SO_3_ group so that OTf^−^ can interact with H_2_O and PC simultaneously, forming [H_2_O‐OTf^−^‐PC] complex.^[^ ^14^ ^]^ Therefore, the introduction of PC could destroy the chemical environment of OTf^−^‐H_2_O in pure Zn(OTf)_2_ electrolyte. As a consequence, PC additives could simultaneously regulate the coordination environment of solvated water and free water in pure Zn(OTf)_2_ electrolyte. In this research, we design different volume ratios between PC and deionized H_2_O to establish the relationship between water coordination environment and proton insertion. The volume ratio of PC to water in mixed electrolytes are 0, 1:4, 1:3, 1:2, 1:1, and the corresponding electrolytes are denoted as PC0, PC14, PC13, PC12, and PC11 (Figure 2e). Due to the high viscosity of PC, the ionic conductivity of mixed electrolyte reduces gradually (Figure S7, Supporting Information). However, the high safety of aqueous electrolytes is inherited by mixed electrolytes (Figure S8, Supporting Information).

### 2.2 Electrolyte Structure Design

**Figure 3:**
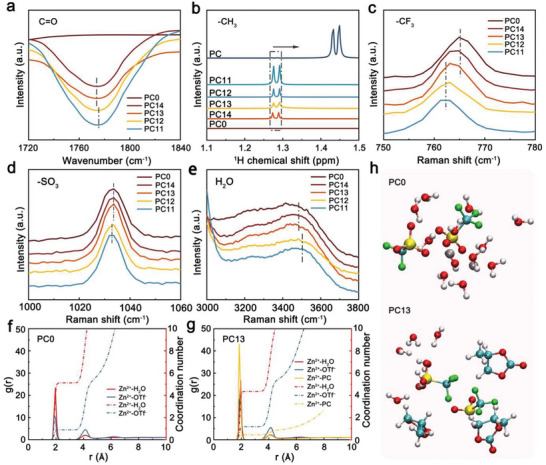
Effect of PC on the coordination environment of electrolyte. a) The FTIR spectra of C═O for all electrolytes. b) The ^1^H NMR spectra of methyl hydrogen of PC for all electrolytes. c–e) The Raman spectra of ‐CF_3_, ‐SO_3_ and H_2_O for all electrolytes. The RDF plots of Zn^2+^ solvation structure for PC0 electrolyte f) and PC13 electrolyte g). h) The representative coordination environment of free water. Gray: H; red: O; blue: C; green: F; yellow: S.

To clearly explore the evolution on coordination environment of electrolytes, Raman, FTIR, and ^1^H NMR spectra were carried out. As shown in **Figure** **3**a, the C═O stretching vibration band gradually shifts to a higher wavenumber with increasing PC contents, indicating that PC molecules tend to enter into Zn^2+^ solvation structure and alter the chemical environment of solvated water.^[^ ^15^ ^]^ In addition, the splitting peaks at 1.43 and 1.44 ppm belonging to methyl hydrogen of PC shifts to the lower field with the addition of PC, showing the enhancement the interactions between ‐CH_3_ of PC molecules and ‐CF_3_ of OTf^−^ anions (Figure 3b). The shifts of ‐CF_3_ and ‐SO_3_ stretching vibration bands also prove it (Figure 3c,d). The existence of PC also decreases the strong H‐bond ratios of electrolytes as displayed by the blue shift of the O‐H stretching mode, proving that the changes on the coordination environment of water (Figure 3e; Figure S9, Supporting Information).^[^ ^10^ ^]^ In addition, the ^1^H chemical shift in H_2_O gradually shifting to higher field with the addition of PC suggests that the electron density on ^1^H from H_2_O gradually decreases, demonstrating the decreased H‐bond interaction in water (Figure S10, Supporting Information).^[^ ^16^ ^]^ The above results confirm that the coordination environment of water molecules has been obviously altered after introducing PC.

Molecular dynamics (MD) simulations were further utilized to analyze the water molecules environment of electrolytes in detail (Figure S11, Supporting Information). From the results of radial distribution function (RDF), H_2_O and OTf^−^ closely involve in the Zn^2+^ primary solvation shells in PC0 electrolyte (Figure 3f). With increasing PC contents, PC molecules gradually replace water molecules of Zn^2+^ primary solvation sheath in mixed electrolytes while the variation on the contribution of OTf^−^ involving in Zn^2+^ primary solvation sheaths could be ignored (Figure 3g; Figure S12, Supporting Information). Further details about the variation of Zn^2+^ solvation structure is shown in Table S2 (Supporting Information). In addition, the result of RDF also shows the changes on chemical environment of OTf^−^ with adding PC (Figure S13, Supporting Information). For representative coordination structures of electrolytes, OTf^−^ anions are only coordinated with water molecules in PC0 electrolyte while OTf^−^ anions are surrounded by H_2_O and PC molecules constructing the hydrogen‐bond network of [H_2_O‐OTf^−^‐PC] complex in mixed electrolytes (Figure 3h; Figure S14, Supporting Information).^[^ ^14^, ^16^ ^]^ Combining these results, PC molecules could regulate water coordination environment in electrolyte by involving in Zn^2+^ solvation structure and forming [H_2_O‐OTf^−^‐PC] complex.

### 2.3 The Relationship Between Proton Insertion and Water Coordination Environment

**Figure 4:**
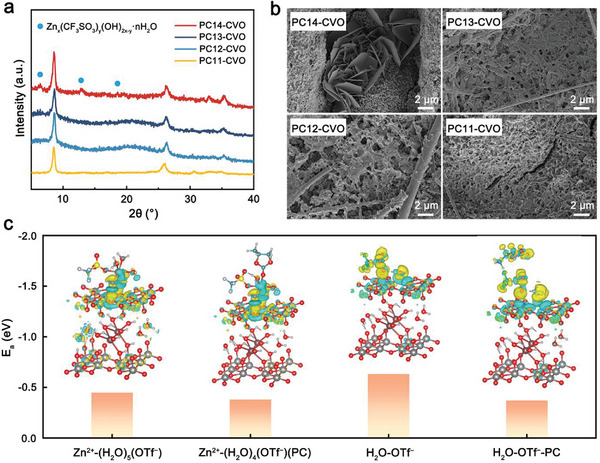
The characterizations of proton insertion and the adsorption geometry of electrolytes on the CVO cathode surface. The XRD patterns a) and SEM images b) of discharged CVO electrode with different electrolytes after 2 cycles at 0.2 A g^−1^. c) The optimized adsorption geometry of water molecules on the electrode surface.

Zn||CVO batteries were assembled with PC14/PC13/PC12/PC11 electrolytes (named PC14‐CVO, PC13‐CVO, PC12‐CVO and PC11‐CVO) to evaluate the electrochemical behavior of proton insertion with the variation in the coordination environment of water molecules in electrolytes. As shown in **Figure** **4**a, when the volume ratio of PC to H_2_O is 1:4, we can observe the weak diffraction peak belonging to BZS. As increasing the content of PC, only the diffraction peaks of CVO are detected. Moreover, sheet‐like by‐products are only observed on discharged PC14‐CVO electrode (Figure 4b). From the TEM element mapping images and EDS results, only PC14‐CVO possesses strong S signal accorded with the composition of BZS (Figure S15–S18, Supporting Information). The results verify that proton insertion is obviously suppressed when the volume ratio of PC to H_2_O is up to 1:3. Based on proton insertion mechanism, we construct an optimized adsorption geometry of water molecules on the CVO electrode surface (Figure S19, Supporting Information). For the solvated water, the Zn^2+^‐(H_2_O)_5.11_·(OTf^−^)_0.88_ for PC0 electrolyte is simplified as Zn^2+^‐(H_2_O)_5_·(OTf^−^), and Zn^2+^‐(H_2_O)_4.35_·(OTf^−^)_1.17_(PC)_0.44_ for PC13 electrolyte is simplified as Zn^2+^‐(H_2_O)_4_·(OTf^−^)(PC). For free water, the structures in PC0 and PC13 electrolyte are simplified as H_2_O‐OTf^−^ and H_2_O‐OTf^−^‐PC, respectively. The adsorption energy of the Zn^2+^‐(H_2_O)_5_·(OTf^−^) and H_2_O‐OTf^−^ at the vanadium oxides surface is determined as −0.448 and −0.631 eV for PC0 electrolyte, while that of Zn^2+^‐(H_2_O)_4_·(OTf^−^)(PC) and H_2_O‐OTf^−^‐PC is only −0.380 and −0.370 eV, respectively (Figure 4c). It is clear that the optimized coordination environment of water decreases the adsorption energy between water molecules and vanadium oxides surface, thus inhibiting proton insertion.

### 2.4 The Relationship Between Proton Insertion and ZVO

**Figure 5:**
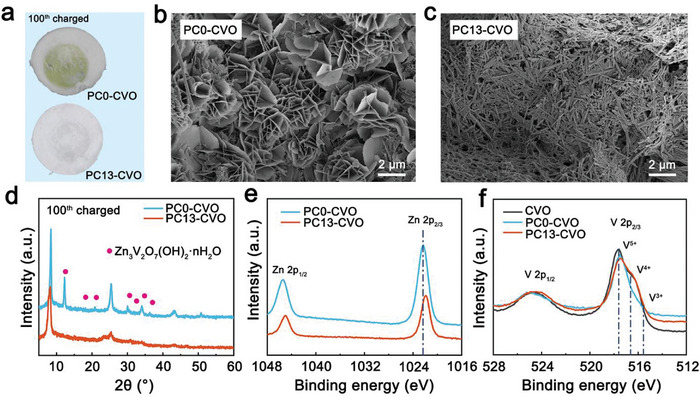
The structural and morphological analyses of charged CVO electrode after 100 cycles at 0.2 A g^−1^. a) The photos of the separator for charged CVO electrode after 100 cycles. The SEM images b,c) and XRD patterns d) of charged CVO electrodes with PC0 and PC13 electrolyte. XPS spectra of PC0‐CVO and PC13‐CVO for Zn 2p e), and V 2p f).

We evaluate the effect of inserted charge carriers on the stability of CVO structure after 100 cycles at 0.2 A g^−1^. As shown in **Figure** **5**a, the color of separator from battery based on PC0 electrolyte becomes yellow due to the dissolution of CVO to form VO_2_(OH)_2_^−^.^[^ ^14^, ^16^ ^]^ However, there is no obvious color change for the separator from the battery based on PC13 electrolyte. The above‐mentioned soaking experiment has proved that the CVO could not dissolve in PC0 electrolyte. Therefore, we can deduce that H^+^ insertion may cause the dissolution of V into electrolyte. Moreover, VO_2_(OH)_2_^−^ will reacts with Zn^2+^ to form ZVO (Zn_3_V_2_O_7_(OH)_2_·nH_2_O). For ZVO phase, Zn atoms occupy the octahedral sites in a close‐packed layer of O atoms, which would constrain Zn^2+^/H^+^ migration and there are not additional active sites for ion insertion.^[^ ^13c^ ^]^ Therefore, the production of electrochemically inactive ZVO is not conducive to electrochemical performance. As shown in Figure 5b,c, numerous nanoflower‐like by‐products accumulate on the surface of PC0‐CVO electrode while PC13‐CVO electrode still maintains the same nanorod‐like morphology as pristine CVO. From TEM element mapping images of nanoflower‐like by‐products at charged PC0‐CVO electrode, strong Zn, V and O signals are detected demonstrating that the nanoflower‐like by‐products would belong to ZVO (Figure S20, Supporting Information).

The by‐products were further investigated by XPS and XRD to demonstrate the existence of ZVO on the PC0‐CVO electrode surface. The peaks of PC0‐CVO at 12.38°, 16.86°, 20.78°, 30.11°, 31.88°, 34.09°, 36.30° in XRD patterns are well indexed to ZVO (Figure 5d).^[^ ^13^, ^17^ ^]^ In contrary, the diffraction peak of PC13‐CVO matches well with pristine CVO without any impurity phase. Moreover, in the Zn 2p spectra, the PC0‐CVO (1022.3 eV) possesses higher binding energy compared with PC13‐CVO (1021.9 eV) due to the existence of ZVO (Figure 5e). Moreover, the V valence state of PC0‐CVO performs +5 with ZVO covering the electrode surface (Figure 5f). By contrary, the V valence state of charged PC13‐CVO remains consistent with that of pristine CVO after 100 cycles. Above results clearly demonstrate that inhibiting proton insertion can avoid the continuous dissolution of vanadium oxide to produce ZVO during on cycling process.

### 2.5 Electrochemical Performance of Zn||CVO Battery

**Figure 6:**
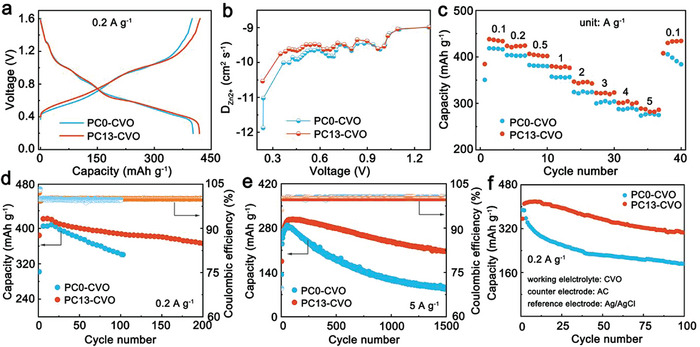
Electrochemical performance of Zn||CVO battery. a) The first charge‐discharge profiles of PC0‐CVO and PC13‐CVO. b) The ion diffusion coefficient during discharging process of the fourth cycle. c) The rate capability of PC0‐CVO and PC13‐CVO. d,e) Cycling performance and coulombic efficiency of PC0‐CVO and PC13‐CVO at 0.2 and 5 A g^−1^. f) The cycle performance of three electrode cell with PCO/PC13 electrolytes at 0.2 A g^−1^.

After inhibiting proton insertion, PC13‐CVO performs enhanced electrochemical performance. As shown in **Figure** **6**a, the discharge capacity of PC13‐CVO is higher than that of PC0‐CVO (418 vs 402 mAh g^−1^) at 0.2 A g^−1^. H^+^ and Zn^2+^ belong to competitive insertion charge carriers. Zn^2+^ also can continuously insert into cathode material after the inhibition of proton insertion. Therefore, PC13‐CVO with only Zn^2+^ insertion can provide near capacity as PC0‐CVO. Moreover, due to the positive effect of PC on the Zn anode, the capacity of PC13‐CVO is slightly higher than that of PC0‐CVO. The results of XPS and CV curves also could support it. From the V 2p spectra, the peak position of PC13‐CVO shifts toward a lower binding energy compared to that of PCO‐CVO after discharging (Figure S21, Supporting Information). PC13‐CVO also possesses larger CV areas comparing to PC0‐CVO (Figure S22, Supporting Information). We utilize the Galvanostatic Intermittent Iitration Technique (GITT) to study the ion diffusion behavior in discharge process (Figure 6b; Figure S23, Supporting Information).^[^ ^18^ ^]^ As reported in previous researches, proton insertion occurs at the low voltage plateau in Zn(OTf)_2_ electrolyte.^[^ ^19^ ^]^ The ex situ XRD of PC0‐CVO at different discharged state proves that proton inserts into CVO at below 0.6 V in PC0 electrolyte (Figure S24, Supporting Information). Therefore, the ion diffusion coefficient of PC13‐CVO and PC0‐CVO is equivalent in high voltage section owing to that both samples only exist in Zn^2+^ insertion in initial discharge process. And the diffusion coefficient of PC0‐CVO is obviously lower than that of PC13‐CVO when the discharge voltage is below to 0.6 V due to the formation of BZS. In addition, the initial charge transfer impedance of the PC0‐CVO and PC13‐CVO is similar (Figure S25a, Supporting Information). However, due to the formation of ZVO adhering to the PC0‐CVO electrode surface, the charge transfer impedance of PC0‐CVO is larger than that of PC13‐CVO after 100 cycles (Figure S25b, Supporting Information). As a result, PC13‐CVO displays better rate capability than PC0‐CVO. As shown in Figure 6c, PC13‐CVO exhibits higher discharge capacity than PC0‐CVO at low/high current density (433 vs 417 mAh g^−1^ at 0.1 A g^−1^, 287 vs 276 mAh g^−1^ at 5 A g^−1^). Moreover, when the current density returns back to 0.1 A g^−1^, the capacity of PC13‐CVO can recover to 433 mAh g^−1^. However, the corresponding capacity of PC0‐CVO is only 398 mAh g^−1^. In addition, due to the interference of BZS and ZVO on PC0‐CVO electrode, PC0‐CVO shows higher self‐discharge rate (η, 10.1%) than PC13‐CVO (η, 3.9%) (Figure S26, Supporting Information).

From the cycling performance, PC13‐CVO also displays high discharge capacity retention (≈86%) with the coulombic efficiency of ≈100% after 200 cycles at 0.2 A g^−1^ (Figure 6d). In contrast, PC0‐CVO shows a fast capacity decay after 100 cycles. Even at the high current density of 5 A g^−1^, PC13‐CVO still performs better cycling performance than PC0‐CVO (Figure 6e). To confirm that if PC inserts into CVO acting as pillars to improve cycling performance. We have detected the O 2p and C 1s spectra of discharged CVO electrode after 2 cycles in Figure S27 (Supporting Information). It found that the peak position of PC0‐CVO and PC13‐CVO keeps identical without C═O peak belonging to PC, indicating that PC molecular does not insert in CVO cathode. In addition, PC additives can relieve HER and the production of insulating byproducts on Zn anode, improving the lifetime of Zn||Zn cells.^[^ ^14^ ^]^ The improvement on Zn anode is also beneficial for cycling performance of Zn||CVO batteries. Therefore, the effect of proton insertion on cycle life of CVO cathode is further investigated by a three‐electrode cell using PC0/PC13 as the electrolyte, saturated Ag/AgCl electrode as the reference electrode, CVO as the working electrode, and AC as the counter electrode to eliminate the effect of the Zn anode (Figure S28, Supporting Information).^[^ ^20^ ^]^ As shown in Figure 6f, the PC13‐CVO still performs obvious enhanced cycling performance comparing to PC0‐CVO. Moreover, from the XRD, the ZVO phase is also detected on the charged PC0‐CVO electrode, while the PC13‐CVO maintains pure CVO phase after 100 cycles (Figure S29, Supporting Information). In summary, the comparison of electrochemical performance between PC0‐CVO and PC13‐CVO demonstrates that inhibiting proton insertion is beneficial for avoiding the dissolution of vanadium oxide to produce ZVO and enhancing cycling performance (Table S3, Supporting Information).

## 3 Conclusion

We demonstrate that proton insertion plays a negative role on the lifetime of vanadium‐based oxides cathode. In Zn(OTf)_2_ electrolyte, water molecules existing in the form of H_2_O‐OTf^−^ and Zn^2+^‐(H_2_O)_5_·(OTf^−^) have strong adsorption energy with vanadium oxide cathode, which drives proton insertion into cathode, while the remaining OH^−^ accumulates on the cathode‐electrolyte interface producing BZS. The proton insertion leads to the dissolution of vanadium oxides to produce electrochemically inactive ZVO. After introducing PC to regulate the free water and solvated water in electrolyte simultaneously, the formed [H_2_O‐OTf^−^‐PC] complex and PC‐coordinated Zn^2+^ solvation structure perform weak interaction with vanadium oxides, thus suppressing the proton insertion. Without proton participating in electrochemical process, the Zn||CVO battery with PC‐optimized electrolyte displays enhanced capacity, rate capability and cycling performance. This work provides an electrolyte regulation strategy to suppress proton insertion and thus enhance cycling performance of vanadium oxides, which is expected to serve as a stepping‐stone toward advancing aqueous zinc battery to becoming a reliable option for large‐scale energy storage systems.

## Conflict of Interest

The authors declare no conflict of interest.

## Author Contributions

F.W. and X.G. conceived the idea. C.Z. performed the experiments. L.Z. and H.R. contributed to electrochemical measurement. M.Y. and M.X. analyzed the FITR and Raman results. T.C. assisted in molecular simulation. F.W., X.G. and C.Z. wrote the paper. All authors took part in the result discussion and data analysis.

## Supporting information



Supporting Information

## Data Availability

The data that support the findings of this s are available from the corresponding author upon reasonable request.
