# Supplementary material for: The Negative Role of Proton Insertion on the Lifetime of Vanadium‐Based Aqueous Zinc Batteries
Source: Adv Sci (Weinh). 2025 Jan 31;12(12):2414762. doi: 10.1002/advs.202414762 (PMC11948006; doi:10.1002/advs.202414762)
Supplement: Supplementary file 1 — Supporting Information [file ADVS-12-2414762-s001.docx]

**Supporting Information**

**The Negative Role of Proton Insertion on the Lifetime of Vanadium-based Aqueous Zinc Batteries**

*Chaoqiong Zhu, Limin Zheng, Hao Ruan, Meng Xiao, Meng Ye, Ting Chen, Fang Wan*, and Xiaodong Guo**

**Materials and Methods**

**Preparation of CaV_8_O_20_·nH_2_O**: CaV_8_O_20_·nH_2_O nanoribbons were prepared by a simple hydrothermal method. 0.81 g V_2_O_5_ (aladdin, >99%) and 0.24 g CaCO_3_ (aladdin, >99%) are dispersed in 50 mL deionized water to form evenly mixed solution with stirring for 30 min. Then, adding CH_3_COOH solution (aladdin, >99.8%) into the mixed solution to adjust pH of solution to 3 and continuing to stir for 30 min. Finally, the mixed solution was placed in a 100 mL Teflon-lined stainless-steel autoclave and hydrothermally treated at 180 °C for 72 h. The product was washed with water and ethanol for several times, and then dried in vacuum oven at 80 °C for 12 h.

Assemble of Zn||CaV_8_O_20_·nH_2_O (CVO) batteries: Cathode material, conductive agent (super p), and PVDF with the weight ratio of 7:2:1 mixed with N-methyl pyrrolidinone (NMP) to form uniform slurry by ball milling for 40 min. The obtained slurry was coated on stainless steel mesh (Φ10 mm) and dried at 80°C overnight. The loading of active cathode material was fixed at ~1.5 mg cm^-2^. 2025-coin cells were assembled with cathode, separator (glass fiber, Φ16 mm), electrolyte (~120 μL) and Zn metal anode (Φ12 mm, thickness (50 µm)).

**Assemble three-electrode cell:** The AC powders, super P and PVDF with the weight ratio of 7:2:1 mixed with NMP to form uniform slurry by ball milling for 40 min. The obtained slurry was coated on carbon cloth (Φ12 mm) and dried at 60°C overnight, preparing AC electrode. The loading of AC was fixed at ~20 mg cm^-2^. The three-electrode cells (swagelok cells) are assembled with CVO as the working electrode cathode, aturated Ag/AgCl electrode as the reference electrode, AC as the counter electrode, electrolyte (electrolyte needs to immerse the reference electrode, ~ 300 μL) and separator (glass fiber, Φ12 mm).

**Characterization:** The ionic conductivity of electrolytes was tested by Ionic conductivity meter (DDS-307). The morphology of CVO was characterized by SEM (ZEISS GeminiSEM 300) and TEM (JEM-F200, 80 kV). The X-ray diffraction (XRD) patterns of the samples were recorded by PW 1730 (Philips Company) with Cu Kα radiation. Raman, FTIR and XPS spectra were collected through Renishaw inVia (523 nm), Thermo Scientific Nicolet iS5 and Thermo Scientific K-Alpha, respectively. The V content was test by ICP-OES with Agilent 5110(OES). ^1^H NMR was performed by Bruker 400MHz. CV curves were measured by an electrochemical workstation (CHI 660E) with a voltage window of 0.2-1.6 V at 0.2 mV s^-1^. EIS were tested under an electrochemical workstation (CHI 660e) with specify frequencies (high frequency: 100 KHz, low frequency: 100 mHz). GITT was performed on a battery test system (LAND CT2001A) with a voltage range from 0.2 to 1.6 V and current pulse of 50 mA g^-1^ was applied for 20 minutes and the followed relaxation time was 120 minutes. Galvanostatic charge/discharge tests were performed on a battery test system with a voltage range from 0.2 to 1.6 V.

**Computational Methods:** The molecular dynamics simulations were performed using the GRMOACS 2020.6 package ^[1]^. The Visualization of structures were performed by VMD software ^[2]^. The molecular were mixed in a cubic box with periodic boundary conditions by using PACKMOL package ^[3]^. The Amber Force Field ^[4]^ was selected in this work, which is good for investigation of various small organic molecules ^[5]^. The ACPYPE ^[6]^ code was used to generate the desired force field parameters for the Sorbitol and Choline chloride. Before starting MD simulation, the initial conFigurations were relaxed using a conjugate gradient minimization scheme. The step size was 0.01 nm, and the cycle was set to 5000 steps. The minimization was considered to have converged when the minimum force was less than 50 kJ·mol^-1^·nm^-1^. The van der Waals interaction was calculated by the cut-off method, atomic electrostatic interaction was calculated by PME (particle mesh Ewald), and both the cut-off and PME distances were 1.0 nm ^[7]^. Then, the system was equilibrated with a pressure of 1.0 bar to achieve a desired density. The Berendsen and V-rescale methods were used to control the pressure and temperature. The time constant was 1.0 ps, and the compressibility was 4.5×10^-5^ bar^-1^. The equilibrium was 5 ns for all systems with a 0.001 ps time step. Finally, the production ran for 50 ns. The pressure control was changed to the Parrinello-Rahman method in the production run. In addition, the LINCS (Linear Constrain Solver) algorithm^[8]^was used to impose constraints on the hydrogen bond.

The Density function theory (DFT) calculations were implemented by using Vienna ab-initio simulation package (VASP). The Perdew-Burke-Ernzerhof (PBE) with generalized gradient approximation (GGA) was employed for the exchange-correlation functional. A plane wave basis with the cutoff energy of 400 eV was chosen in this work. A 5 × 5 × 1 k-point mesh was used in these calculations. We consider the van der Waals (vdW) interaction using the DFT-D3 method. A vacuum layer along the out-plane direction of 15 Å was constructed to restrain the interactions between adjacent slabs. The residual force and energy convergence thresholds were set to 0.01 eV Å^-1^ and 10-5 eV Å^-1^, respectively.

The adsorption energy (Eads) was defined as follows:

$$E\text{ads}=E\text{total}-E\text{mol}-E\text{base}$$

where $E\text{total}$, $E\text{mol}$_，_ and $E\text{base}$, are the total energy of the adsorption model, the energy of molecule, and the energy of the basement, respectively.


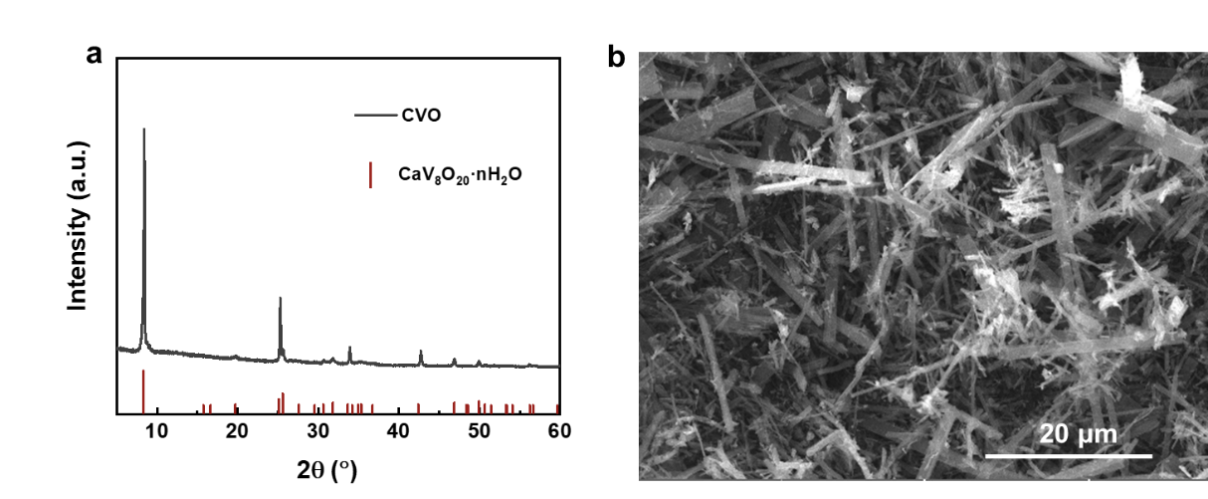


**Figure S1.** The XRD (a) and SEM (b) of the CVO cathode powder.


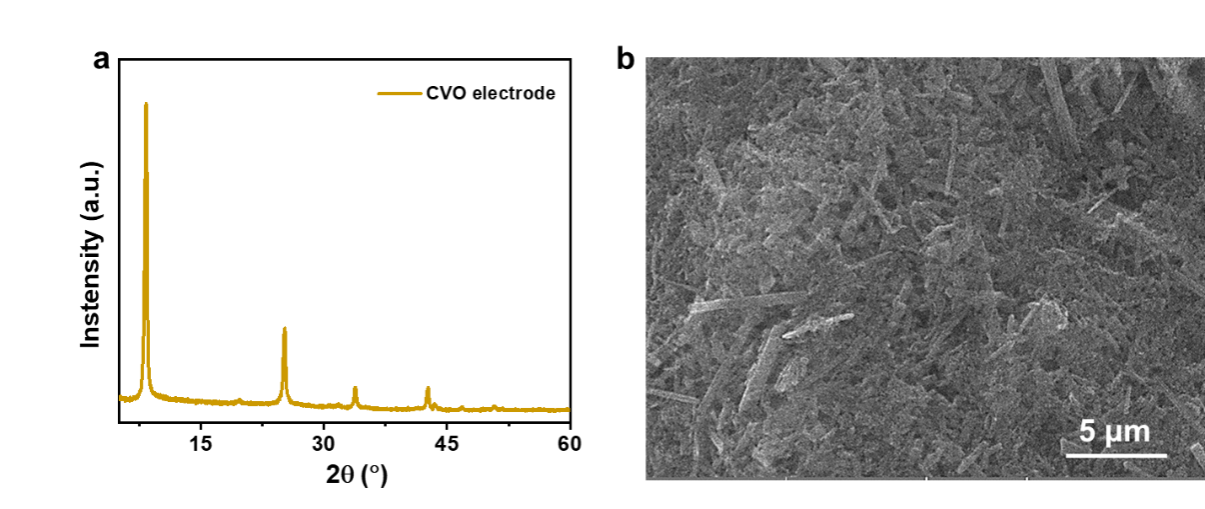


**Figure S2.** The XRD (a) and SEM (b) of the CVO electrode before cycling.


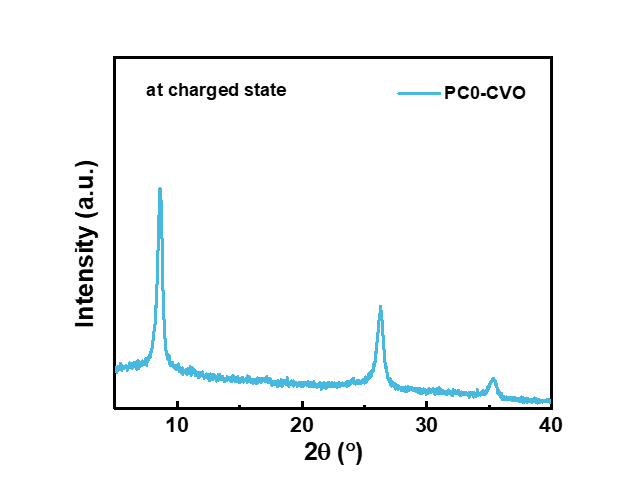


**Figure S3.** The XRD of PC0-CVO at charged state after 2 cycles at 0.2 A g^-1^.


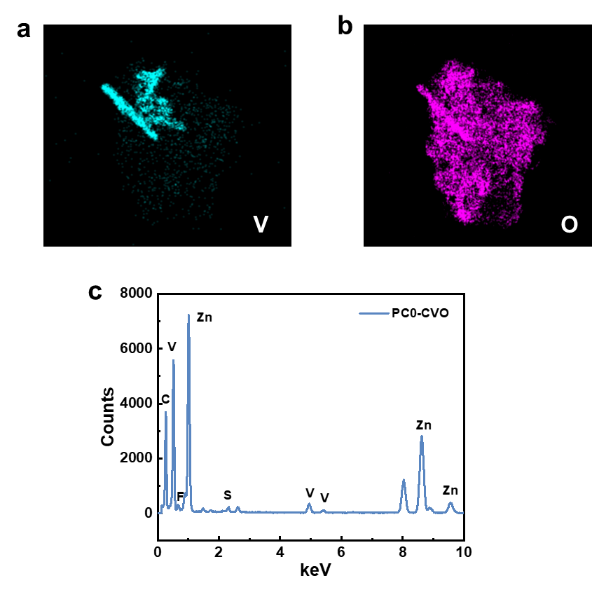


**Figure S4.** The corresponding TEM element mapping images of discharged CVO electrode after 2 cycles at 0.2 A g^-1^.


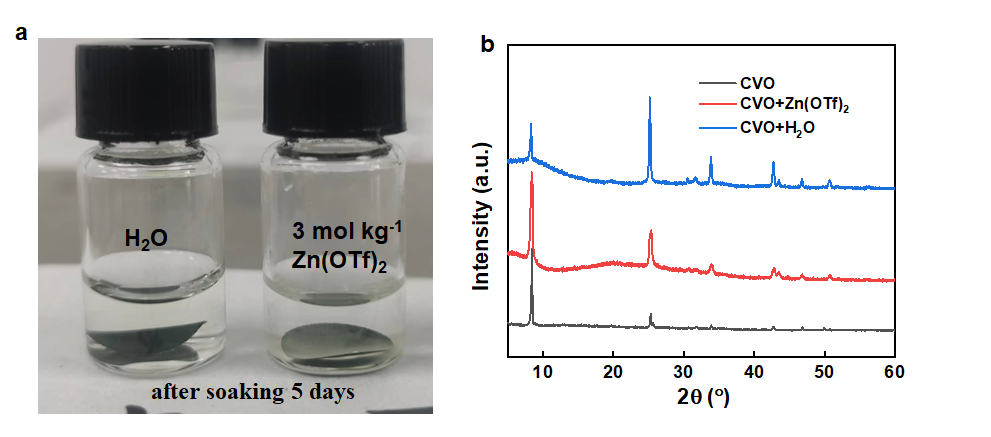


**Figure S5.** (a) The optical photographs of CVO electrodes soaking in H_2_O/Zn(OTf)_2_ solution after 5 days and (b) the corresponding XRD of soaked electrodes.


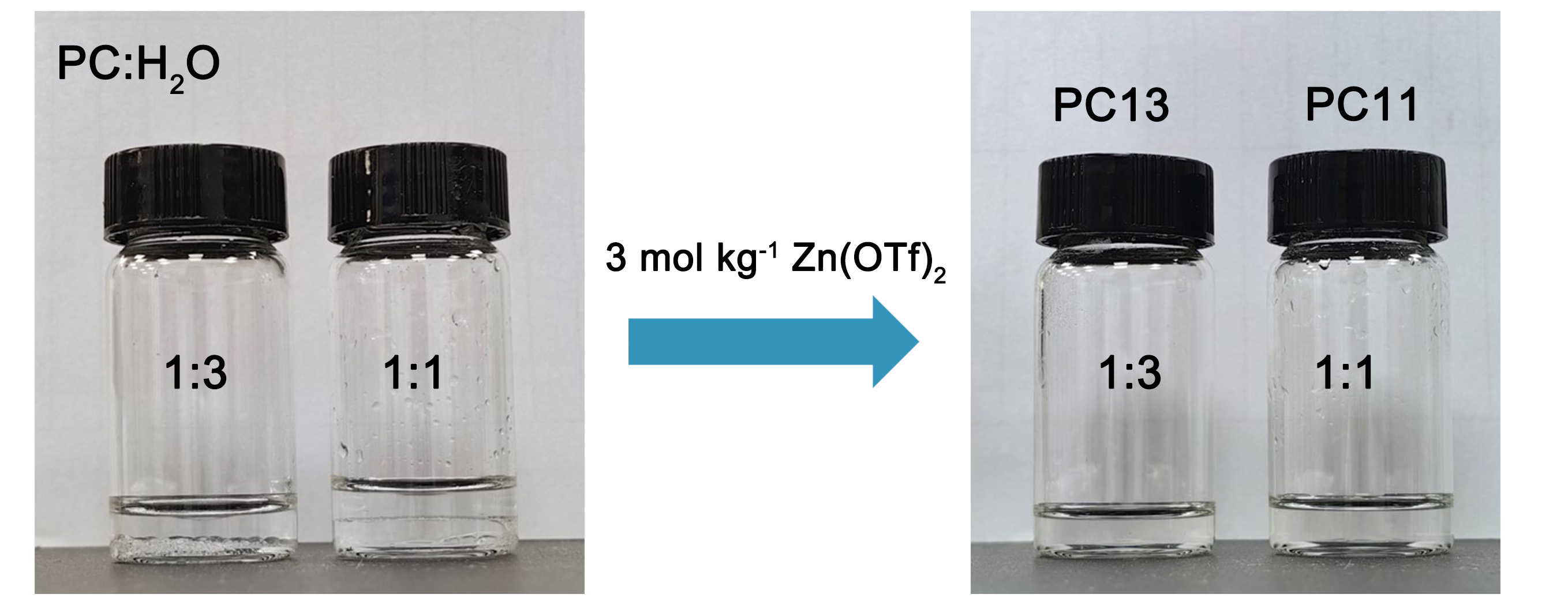


**Figure S6.** Digital photographs of the water/PC mixture with Zn(OTf)_2_ salt.

**Figure S7.** The ionic conductivity of electrolytes.


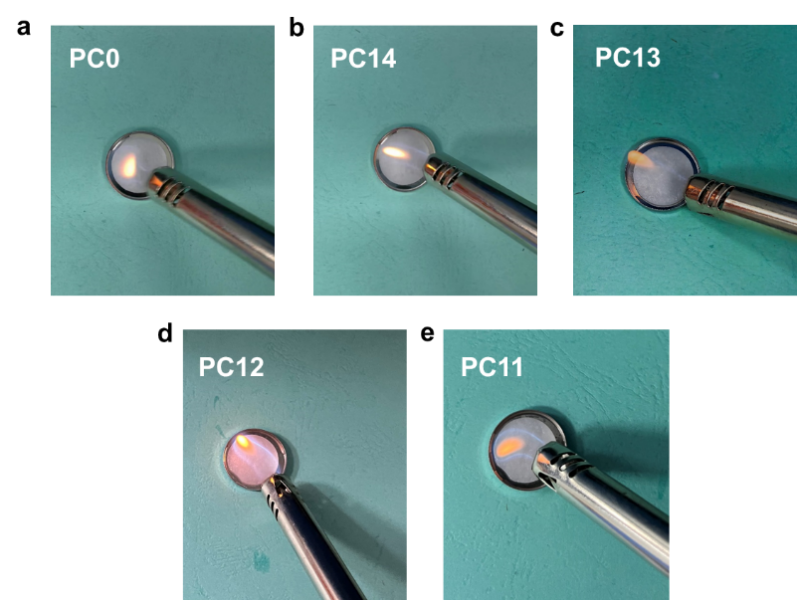


**Figure S8.** The ignition test for the (a) PC0, (b) PC14, (c) PC13, (d) PC12 and (e) PC11.

**Figure S9.** The -OH stretching vibration of all mixed electrolytes.

**Figure S10.** ^1^H NMR spectra of H_2_O for all electrolytes.


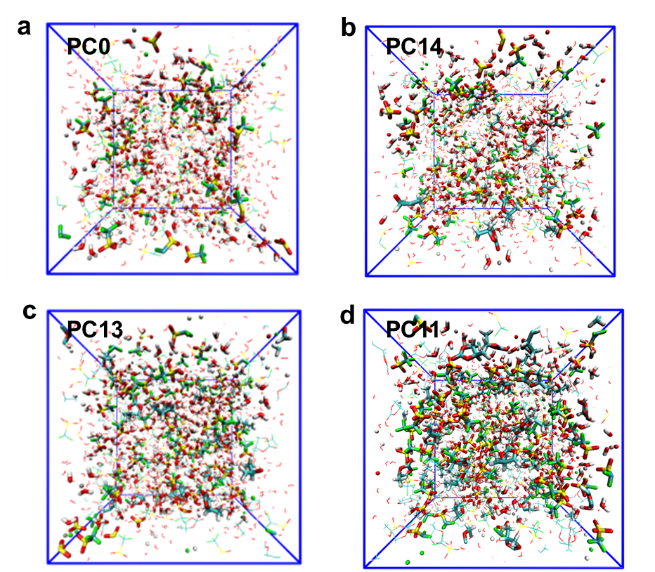


**Figure S11.** The snapshot of the MD simulation cell for PC0, PC14, PC13 and PC11 electrolyte.


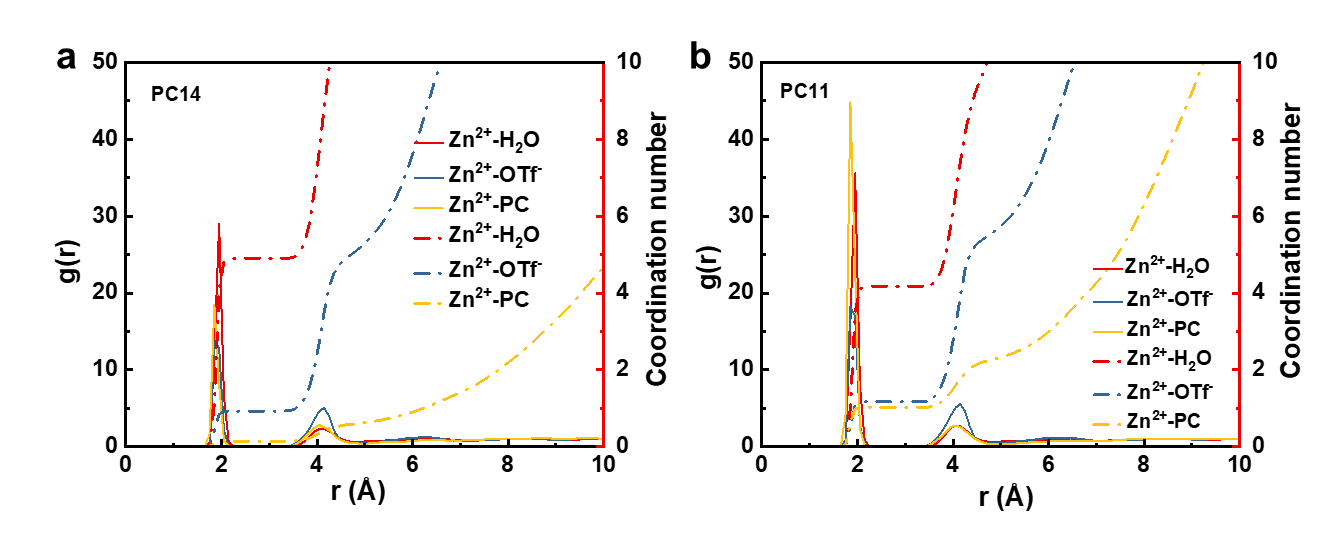


**Figure S12.** The corresponding RDF plots for PC14 and PC11 electrolytes.


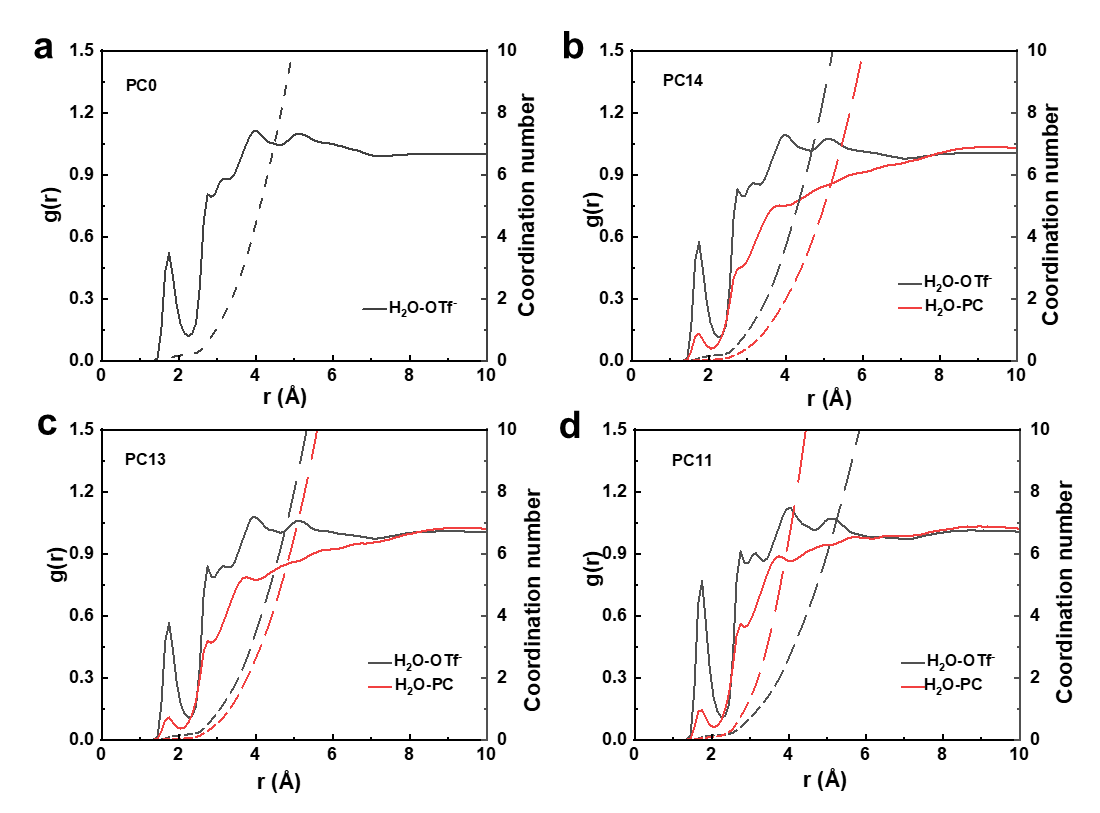


**Figure S13.** The corresponding RDF plots of H_2_O-OTf^-^ and H_2_O-PC for PC0, PC14, PC13 and PC11 electrolytes.


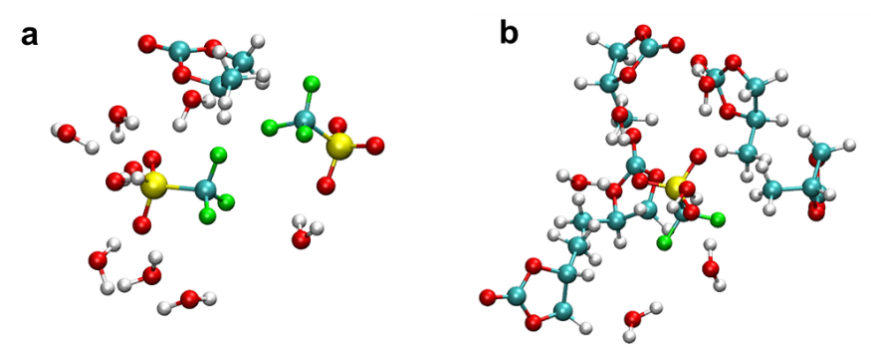


**Figure S14**. The representative coordination environment of free water in PC14 (a) and (b) PC11 electrolytes.


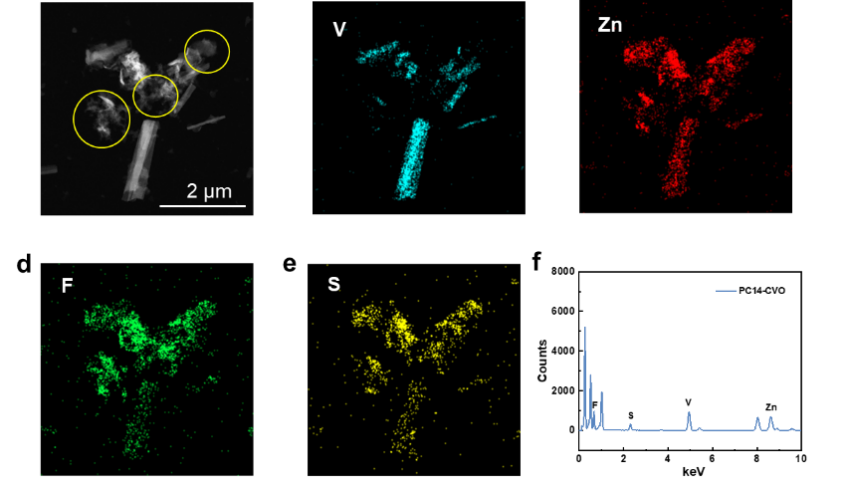


**Figure S15.** (a-f) The corresponding TEM element mapping images of discharged PC14-CVO electrode after 2 cycles at 0.2 A g^-1^.

**
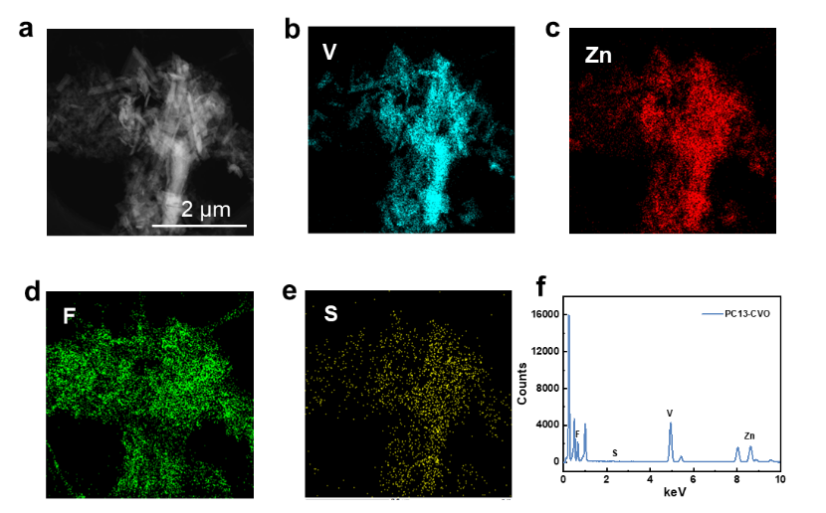
**

**Figure S1****6.** (a-f) The corresponding TEM element mapping images of discharged PC13-CVO electrode after 2 cycles at 0.2 A g^-1^.

**
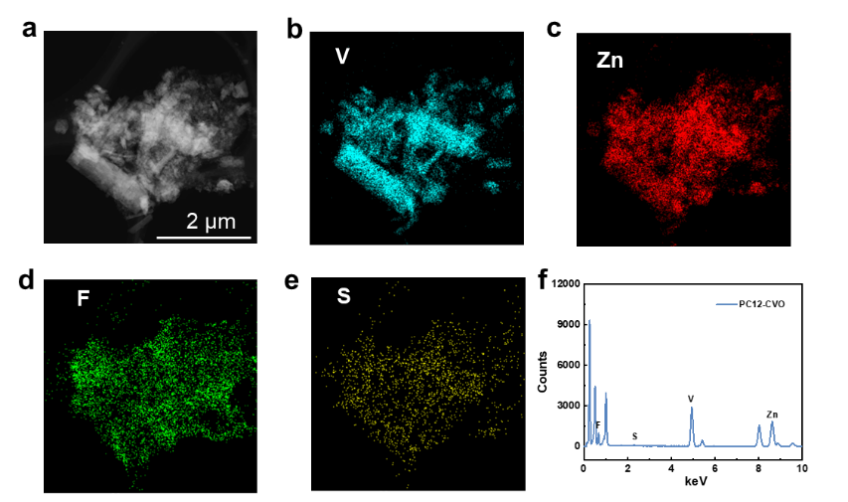
**

**Figure S17.** (a-f) The corresponding TEM images element mapping images of discharged PC12-CVO electrode after 2 cycles at 0.2 A g^-1^.

**
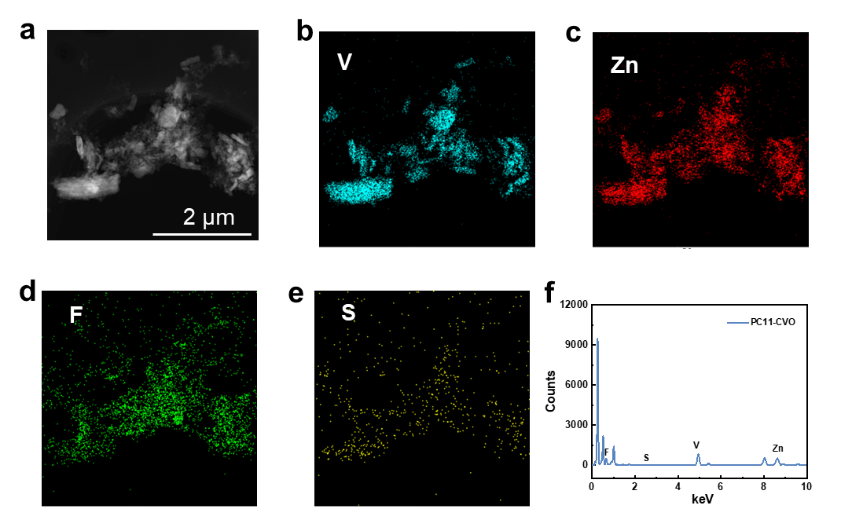
**

**Figure S18.** (a-f) The corresponding TEM images element mapping images of discharged PC11-CVO electrode after 2 cycles at 0.2 A g^-1^.

**
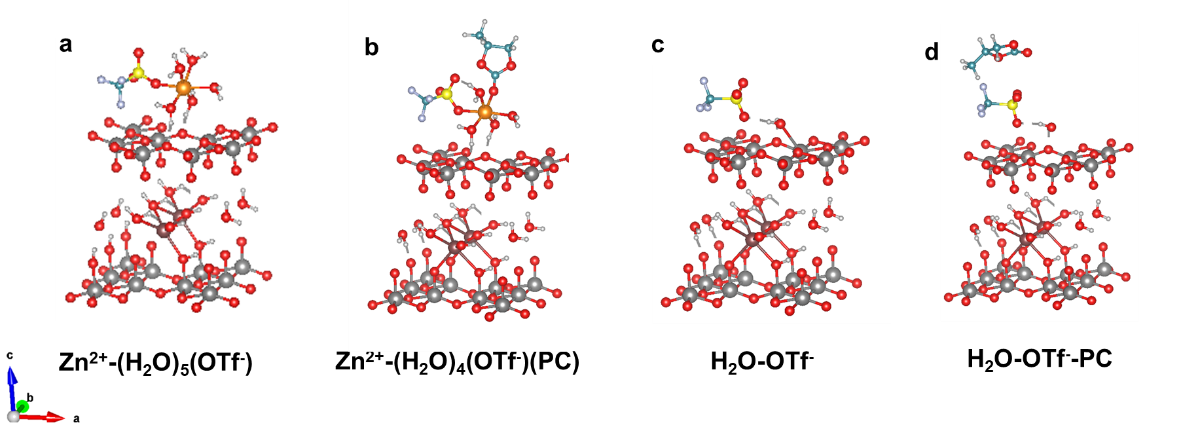
**

**Figure S19.** The optimized adsorption geometry between electrolyte and the surface of CVO electrode.


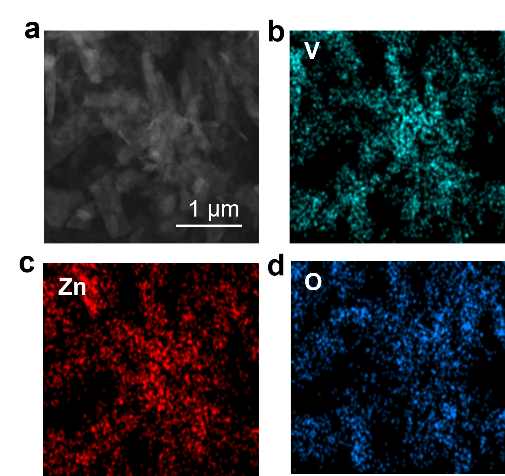


**Figure S20.** The corresponding TEM element mapping images of charged PC0-CVO electrode after 100 cycles at 0.2 A g^-1^.

**Figure S21.** The XPS of V 2p spectra for PC0-CVO and PC13-CVO at fully discharged states.

**
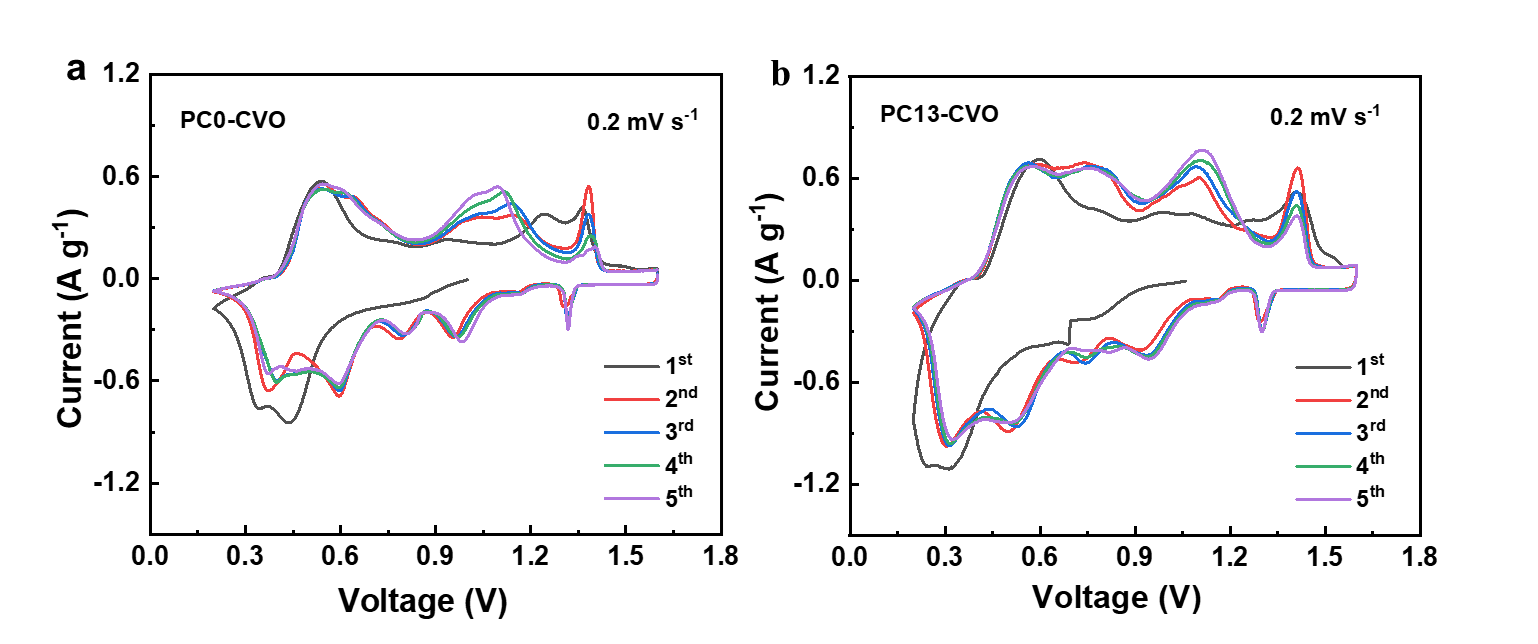
**

**Figure S22.** The CV curves for PC0-CVO and PC13-CVO.

**Figure S23.** The GITT curves of PC0-CVO and PC13-CVO after 4 cycles at 0.05 A g^-1^.

**
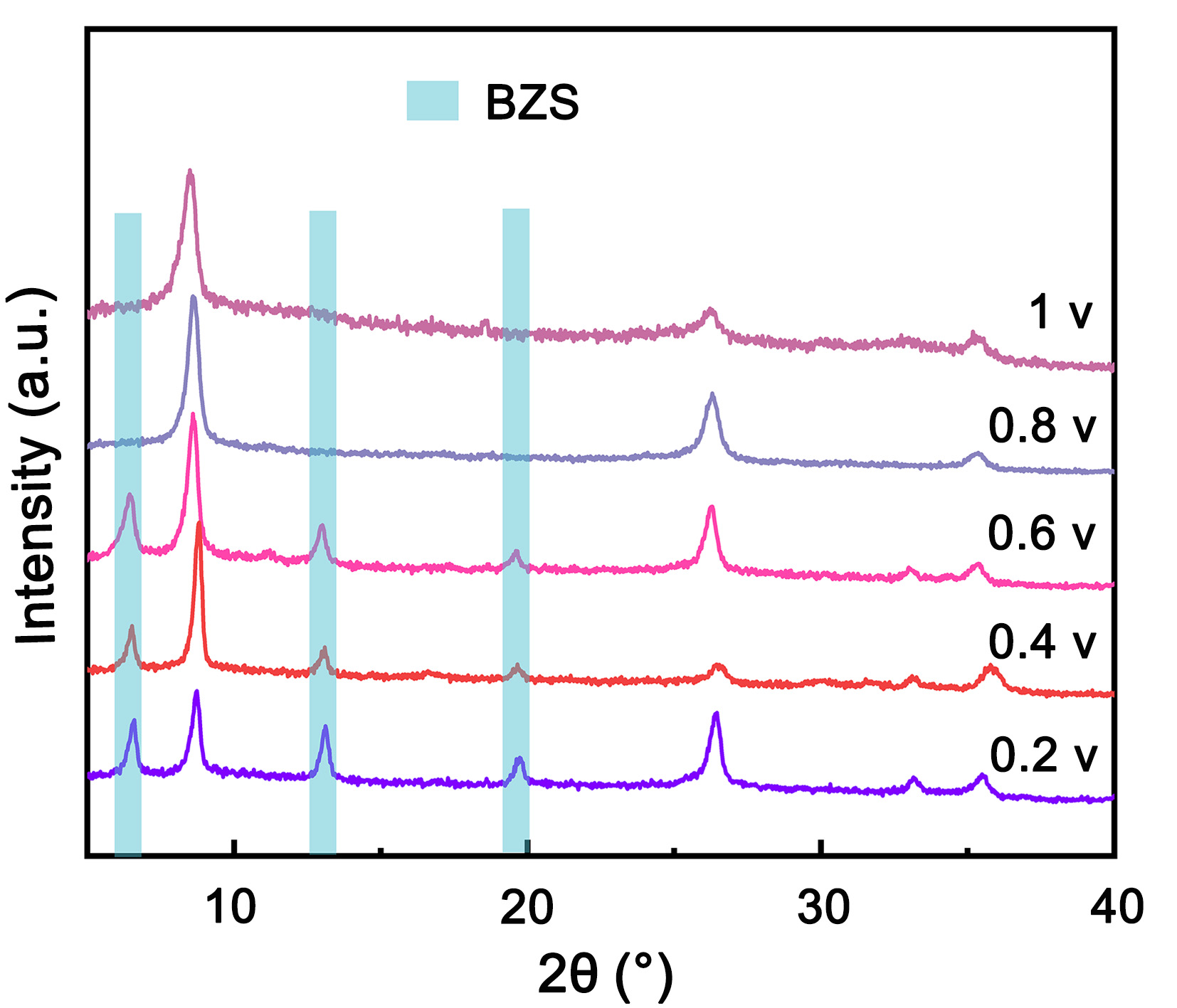
**

**Figure S24.** The ex-situ XRD of PC0-CVO at different discharged state.

**
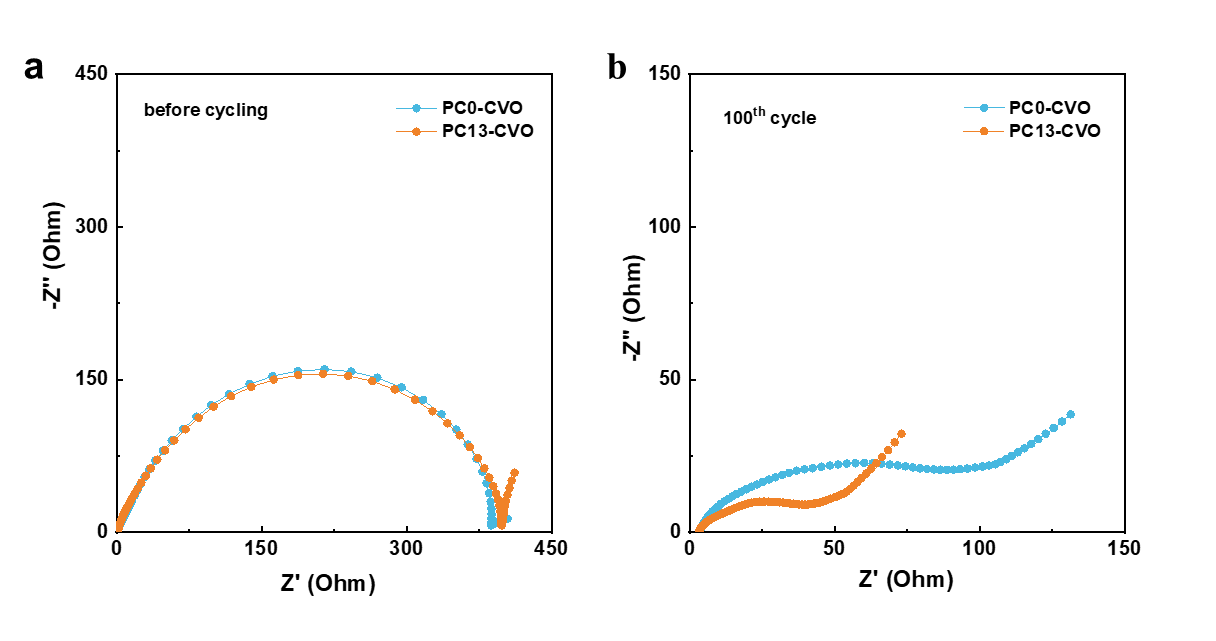
**

**Figure S25.** Electrochemical impedance spectroscopy (EIS) of PC0-CVO and PC13-CVO before cycling (a) and after 100 cycles (b).

**
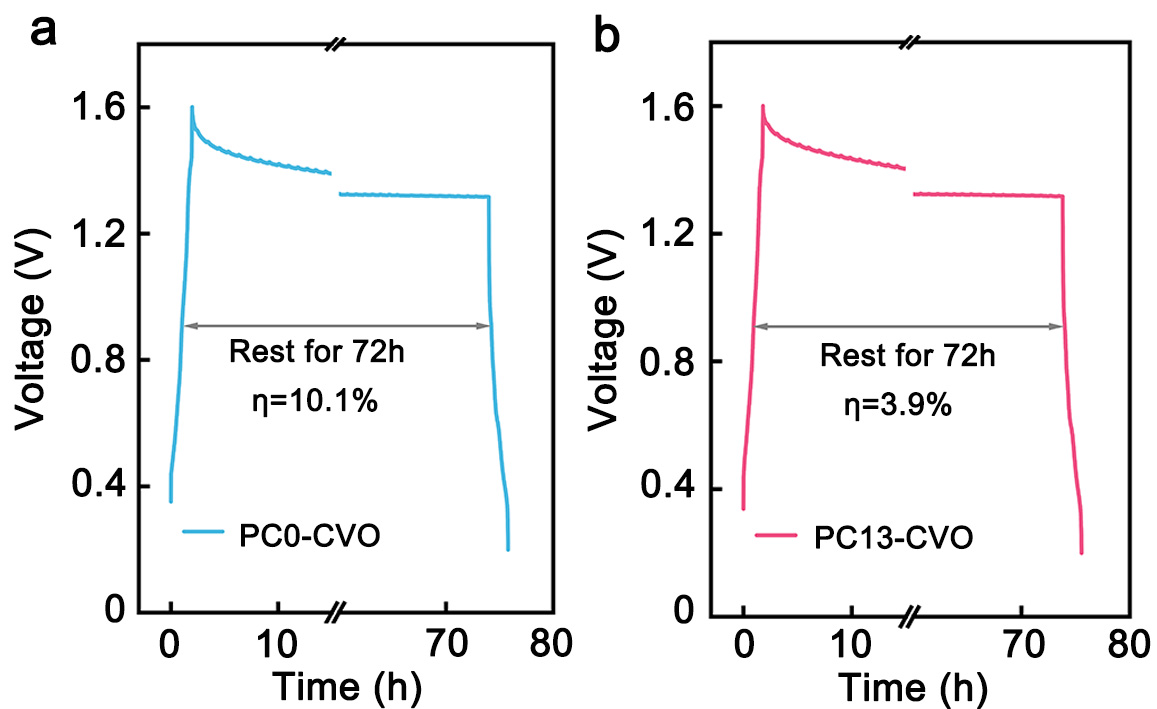
**

**Figure S26.** The battery self-discharge behavior of PC0-CVO (a) and PC13-CVO (b).

**
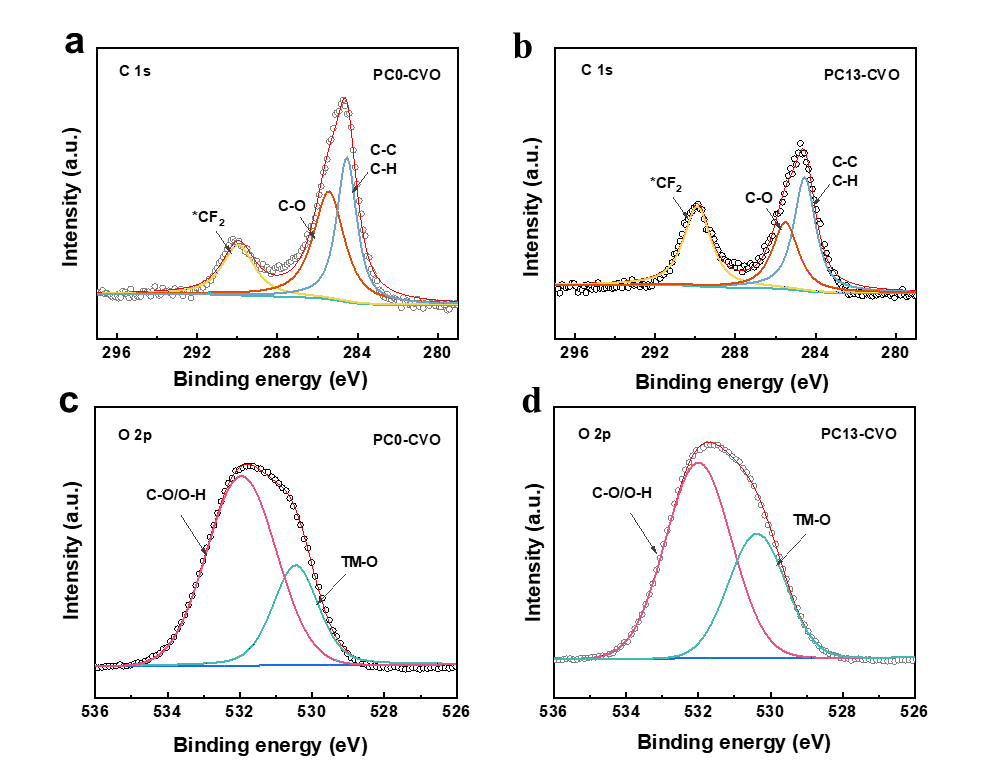
**

**Figure S27.** (a-d) The XPS of etched 10 nm for PC0-CVO and PC13-CVO at fully discharged states after 2 cycles at 0.2 A g^-1^.

**
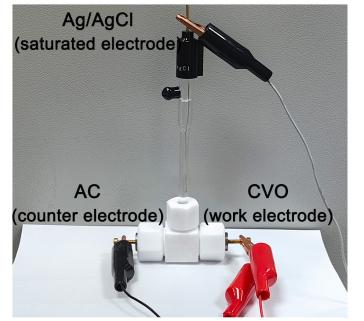
**

**Figure S28.** The digital photo of three electrode cell.

**Figure S29.** The XRD of PC0-CVO and PC13-CVO by a three-electrode cell after 100 cycles at 0.2 A g^-1^.

**Table S1.** V concentration in pure water or Zn(OTf)_2_ electrolyte after soaking CVO electrodes for 2/5 days.

|  | **H_2_O** | | **3M Zn(OTf)_2_** | |
| --- | --- | --- | --- | --- |
| **Soaking time (d)** | 2 | 5 | 2 | 5 |
| **V concentration (mg/L)** | 7.41 | 7.93 | 1.24 | 1.67 |

**Table S2.** Details about Zn^2+^ solvation structure in electrolytes.

|  | **PC0** | **PC14** | **PC13** | **PC11** |
| --- | --- | --- | --- | --- |
| **H_2_O around Zn^2+^** | 5.11 | 4.9 | 4.35 | 4.17 |
| **OTf^-^ around Zn^2+^** | 0.88 | 0.93 | 1.17 | 0.76 |
| **PC around Zn^2+^** | / | 0.14 | 0.44 | 1.02 |

**Table S3.** The Comparison of electrochemical performance between PC0-CVO and PC13-CVO.

|  | **Capacity (Coin cell)** | **Cycle number** | |
| --- | --- | --- | --- |
|  |  | **Coin cell** | **Three electrode cell** |
| **PC0-CVO** | 433, 418, 404, 378, 343, 321, 301, 287 mA h g^-1^ at 0.1, 0.2, 0.5, 1, 2, 3, 4, 5 A g^-1^ | 86% (0.2 A g^-1^， 200 cycles)；  67% (5 Ag^-1^, 1500 cycles) | 86%  (0.2 A g^-1^， 100 cycles) |
| **PC13-CVO** | 417, 402, 381, 355, 321, 302, 287, 276 mA h g^-1^ at 0.1, 0.2, 0.5, 1, 2, 3, 4, 5 A g^-1^ | 83% (0.2 A g^-1^， 100 cycles)；  32% (5 Ag^-1^, 1500 cycles) | 49%  (0.2 A g^-1^， 100 cycles) |

**References**

[1] M. J. Abraham, T. Murtola, R. Schulz, S. Páll, J. C. Smith, B. Hess, E. Lindahl, *SoftwareX* **2015**, *1-2*, 19-25.

[2] William Humphrey, Andrew Dalke, K. Schulten, *J. Mol. Graph.* **1996**, *14*, 33-38.

[3] L. Martinez, R. Andrade, E. G. Birgin, J. M. Martinez, *J. Comput. Chem.* **2009**, *30*, 2157-2164.

[4] J. Wang, R. M. Wolf, J. W. Caldwell, P. A. Kollman, D. A. Case, *J. Comput. Chem.* **2004**, *25*, 1157-1174.

[5] P. Han, W. Nie, G. Zhao, P. Gao, *J. Mol. Liq.* **2022**, *366*, 120243.

[6] A. W. S. d. Silva, W. F. Vranken, *BMC Res. Notes* **2012**, *5*, 367.

[7] P. Li, B. P. Roberts, D. K. Chakravorty, K. M. Merz Jr., *J. Chem. Theory Comput.* **2013**, *9*, 2733-2748.

[8] B. Hess, H. Bekker, H. J. C. Berendsen, J. G. E. M. Fraaije, *J. Comput. Chem.* **1997**, *18*, 1463-1472.
